# Selective Dicer Suppression in the Kidney Alters GSK3β/β-Catenin Pathways Promoting a Glomerulocystic Disease

**DOI:** 10.1371/journal.pone.0119142

**Published:** 2015-03-23

**Authors:** Anna Iervolino, Francesco Trepiccione, Federica Petrillo, Manuela Spagnuolo, Marzia Scarfò, Daniela Frezzetti, Gabriella De Vita, Mario De Felice, Giovambattista Capasso

**Affiliations:** 1 Biogem, Istituto di Ricerche Genetiche Gaetano Salvatore, Ariano Irpino, Italy; 2 Department of Cardio-Thoracic and Respiratory Science, Second University of Naples, Naples, Italy; 3 Department of Molecular Medicine and Medical Biotechnologies, University of Naples “Federico II”, Naples, Italy; 4 Institute of Genetics and Biophysics A. Buzzati Traverso, CNR Naples, Italy; Aarhus University, DENMARK

## Abstract

Dicer is a crucial enzyme for the maturation of miRNAs. Mutations in the Dicer gene are highly associated with Pleuro Pulmonary Blastoma-Family Dysplasia Syndrome (PPB-FDS, OMIM 601200), recently proposed to be renamed Dicer syndrome. Aside from the pulmonary phenotype (blastoma), renal nephroma and thyroid goiter are frequently part of Dicer syndrome. To investigate the renal phenotype, conditional knockout (cKO) mice for Dicer in Pax8 expressing cells were generated. Dicer cKO mice progressively develop a glomerulocystic phenotype coupled with urinary concentration impairment, proteinuria and severe renal failure. Higher cellular turnover of the parietal cells of Bowman’s capsule precedes the development of the cysts and the primary cilium progressively disappears with cyst-enlargement. Upregulation of GSK3β precedes the development of the glomerulocystic phenotype. Downregulation of β-catenin in the renal cortex and its cytosolic removal in the cells lining the cysts may be associated with observed accumulation of GSK3β. Alterations of β-catenin regulating pathways could promote cystic degeneration as in other models. Thus, miRNAs are fundamental in preserving renal morphology and function. Alteration of the GSK3β/β-catenin pathway could be a crucial mechanism linking miRNA dysregulation and the development of a glomerulocystic disease.

## Introduction

MicroRNAs (miRNAs) are small endogenous non-coding RNA molecules that regulate gene expression at the post-transcriptional level [1]. Dicer, an RNase III-type endonuclease, not only is crucial for the final maturation of miRNAs, but also, as part of the RNA-induced silencing complex (RISC), for targeting and regulating mRNA traslation [2–4]. miRNA activity is essential for development since constitutive KO mice die at 7.5 dpc [5]. cKO models have shown that Dicer is crucial for the proper function of renal cells [6,7] and nephron segments [8], especially during organogenesis [9]. In addition, multiple nephron segment deletion leads to cyst development in adult mice [10].

Mutations of Dicer are critical for the development of the Pleuro Pulmonary Blastoma-Family Dysplasia Syndrome (PPB-FDS, OMIM 601200), a condition that affects children with PPB or their family members. Dicer mutations are highly associated with familiar and sporadic PPB-FDS, and thus the designation “Dicer Syndrome” has been proposed for this condition [11]. Renal nephroma and cystic goiter are the most frequent disorders associated with PPB, together with cystic tumors in other organs [11].

To investigate the molecular mechanisms underlying Dicer-dependent cystogenesis, mice exhibiting Dicer cKO in Pax8 expressing tissues, namely thyroid and kidney, were generated. This mouse model properly resembles the frequent clinical association of goiter and renal nephroma.

Both glomerular-cyst development and interstitial fibrosis induced by knocking down Dicer suggest a morphological pattern similar to other glomerulocystic diseases, such as nephronophthisis and medullary cystic kidney disease [12]. Since glomerulocystic diseases present, at different level, alterations in the Wnt/β-catenin pathway, we address here whether miRNA dysregulation impairs GSK3β/β-catenin regulation, two key elements of this signaling cascade. The Wnt pathway is highly conserved among species and regulates several crucial cellular functions such as proliferation and cellular regeneration.

Here we show that the Dicer cKO induced glomerulocystic phenotype is associated with GSK3β and β-catenin dysregulation. Alterations of miRNAs seem to make the parietal cells of the Bowman capsule, a well known adult renal stem-cells niche [13], more susceptible to proliferation.

## Materials and Methods

### Generation of Dicer^Flox/Flox^;Pax8^Cre/+^ mice

To inactivate the Dicer gene in the kidney, mice expressing Cre recombinase under the control of endogenous Pax8 promoter [14] and Dicer^Flox/Flox^ [15] were bred. Dicer^Flox/Flox^;Pax8^Cre/+^ mice were used as the experimental group, named Dicer cKO, while Dicer^Flox/Flox^;Pax8^+/+^ littermates were used as controls (Ctr). Genotyping and Dicer excision were performed by PCR analysis of tail biopsy and renal cortical tissue, respectively, as shown previously [16]. All the procedures involving animals were conducted as indicated by the Italian Ministry of Health in decree nr 100/2006 of July 10^th^ 2006, according to DL N° 116/27/01/1992. In vivo experiments were approved by the Animal Ethics Committee (CESA) of Biogem (Italy) (ID 2710).

### Experimental study

Dicer cKO and their control littermates at 30 and 50 days after birth were studied (P30, P50). All experiments were conducted on age and gender-matched animals. Mice were housed individually in metabolic cages for 5 days at 23°C with a 12h dark/light cycle. Food and water were offered *ad libitum*. After 4 days of adjustment, physiological parameters were collected on day 5. 24h urine output was collected under mineral oil to prevent evaporation. Urinary osmolality was measured by Osmometer 3320 (Advanced Instrument, Inc). Proteinuria was quantified by Bradford Assay and Albuminuria by SDS-PAGE electrophoresis.

### Immunohistochemistry

Immunohistochemistry was performed as previously described [17]. Briefly, mice were anesthetized by isoflurane and perfused through the abdominal aorta with 4% PFA. Blood and the left kidney were collected before perfusion. The left kidney was used for immunoblotting or PCR, while the right kidney was used for immunohistochemistry.

After embedding in paraffin, 4 μm thick sections were stained with hematoxylin and eosin (Sigma-Aldrich) or Masson Trichrome (Bio-optica), according to the manufacturer’s instructions.

For immunohistochemistry, sections were incubated overnight in xylene and then, progressively, in ethanol solution (99–96–70%). Endogenous peroxidase activity was quenched with 35% H_2_O_2_ in methanol and target retrieval was performed in TEG buffer pH 9.2. Primary antibodies were incubated overnight at 4°C.

The primary antibodies were used: anti-Cleaved Caspase-3 (cat. no. 9661, CST) dilution 1:250, anti-AQP2 (7661AP, kindly provided by Prof. Sebastian Frische) dilution 1:1000, anti-Ki67 (cat. no. 4203–1, Epitomics) dilution 1:500, B1-H^+^ATPasi (kindly provided by Prof. Wagner C.) dilution 1:1000, Lectin from Peanut Peroxidase labeled (cat. no. L7759, Sigma) diluition 1:250. Sections were then incubated with secondary anti-rabbit HRP-conjugated antibody (cat. no. 2016–09, Dako). The chromogenic reactions were carried out with DAB (cat. no. 4170, Kementec Diagnostics), with Hematoxylin as nuclear counterstain. Stained sections were mounted with Eukitt (Bio-optica).

For immunofluorescence analysis the sections were incubated overnight at 4°C with anti-Acetylated Tubulin (cat. no. T6793, Sigma-Aldrich) dilution 1:5000, β-catenin (cat. no. 610153, BD Transduction Laboratories) dilution 1:20, Tamm Horsfall Glycoprotein antibody (cat. no. FZ20C, Europa Bioproducts) diluition 1:1000. For double labeling sections were incubated with anti-AQP2 antibody and, after blocking endogenous biotin (Biotin Blocking System, Dako) with Biotinilated H^+^-ATPase (7659AP). Anti-rabbit conjugated Alexa Fluor 488 and Streptavidin Fluor Alexa 546 were used. Zeiss Axioplan 2 microscope was used for image acquisition. For the staining with the anti-Acetylated Tubulin antibody a Leica TCS SP2 confocal microscope was used.

### Renal clearance studies

Inulin clearance was performed as previously described [18]. Briefly, mice were anesthetized with Inactin (Sigma) 100 mg/kgBW, tracheostomized, placed on a surgical table. The left jugular vein was cannulated with a PE-10 catheter for infusion via a syringe pump (Infusion Pump High Tech, KDS Legato 200). The right carotid artery was catheterized to monitor blood pressure through a blood pressure recorder (BP1 by WPI) and to take blood samples. The bladder was catheterized with a PE-50 tube for urine collection. Then, mice received a constant infusion of FITC-inulin (Sigma) at a rate of 0.15 μl/min/gBW. After 60 min of equilibration, urine samples were collected every 30 min. A total of four urine and blood collections were made. GFR was calculated using standard clearance formula. FITC-Inulin concentrations in plasma and urine were measured by a colorimetric method (EnVision).

### Immunoblotting

Left kidneys were sectioned in Cortex/OSOM, ISOM and IM. Tissues were homogenized with a TissueLyser (Qiagen) in Lysis buffer (Sucrose 0,3M, Imidazole 25mM, EDTA 1mM, PMSF 1mM) with protease and phosphatase inhibitor cocktails (Complete Protease Inhibitor Cocktail, cod. Sc-29130, Santa Cruz; PhosSTOP, Roche). Total protein concentration was measured by Bradford assay (Biorad Protein Assay). SDS-PAGE was performed on NuPage 4–12% Bis-Tris Gel. Proteins were then transferred to PVDF membranes (Invitrolon PVDF, Invitrogen).

The membranes were probed with antibodies anti-Dicer1 (Dana-Farber Molecular Biology core Facilities, corresponding to residues 1385–1405) dilution 1:1000, anti-β-catenin (cat. no. 610153, BD Transduction Laboratories) dilution 1:1000, anti-AQP2 (dilution 1:1000), anti-NKCC2 (dilution 1:10000), anti-mTor (CST 2983; diluition 1:1000), anti Phospho-mTor (Ser2448) (CST 5536; diluition 1:1000) anti-S6 Ribosomal protein (CST 2317; diluition 1:1000), anti-PhosphoS6 Ribosomal protein (Ser235/236) (CST 2211; diluition 1:500), anti-β-Actin (Sigma, n. cat. T6793 dilution 1:20000) and GAPDH (GeneTex 100118; diluition 1:20000). Blots were incubated with HRP conjugated secondary antibodies, according to the species of the primary antibodies (Amersham) and then developed using ECL substrate (Pierce). Image-j was used to quantify single band intensity.

### RNA extraction and q-PCR

One microgram of total RNA, isolated from tissues using TRIzol Reagent (Invitrogen Life Technologies, Carlsbad, CA), was reverse-transcribed by Quantitec reverse transcription kit (Qiagen) according to the manufacturer’s instructions.

The qPCR reaction mixture contained 7 ng of total cDNA, Power PCR Master Mix 16 (Applied Biosystems) and the following primers used at a concentration of 300 nm each:

Dicer1 Fw: TTGATGGGAACGCTAACACA; Rev: GCTCCAGGTTAAACCCATCA;

β-Catenin Fw: atggcttggaatgagactgc; Rev: atgctccatcatagggtcca;

RNA18SFw: CGGCTACCACATCCAAGGAA; Rev: GGGCCTCGAAAGAGTCCTGT.

Reactions were run on a 7900HT system (Applied Biosystems). For each sample, the expression of the genes of interest was normalized for the expression of RNA18S and measured under the same conditions.

### Statistics

Values are represented as mean ± standard errors. Data were analyzed by Student’s *t*-test. P-value < 0.05 was considered significant.

## Results

### Genotyping of Dicer cKO mice

**Fig 1:**
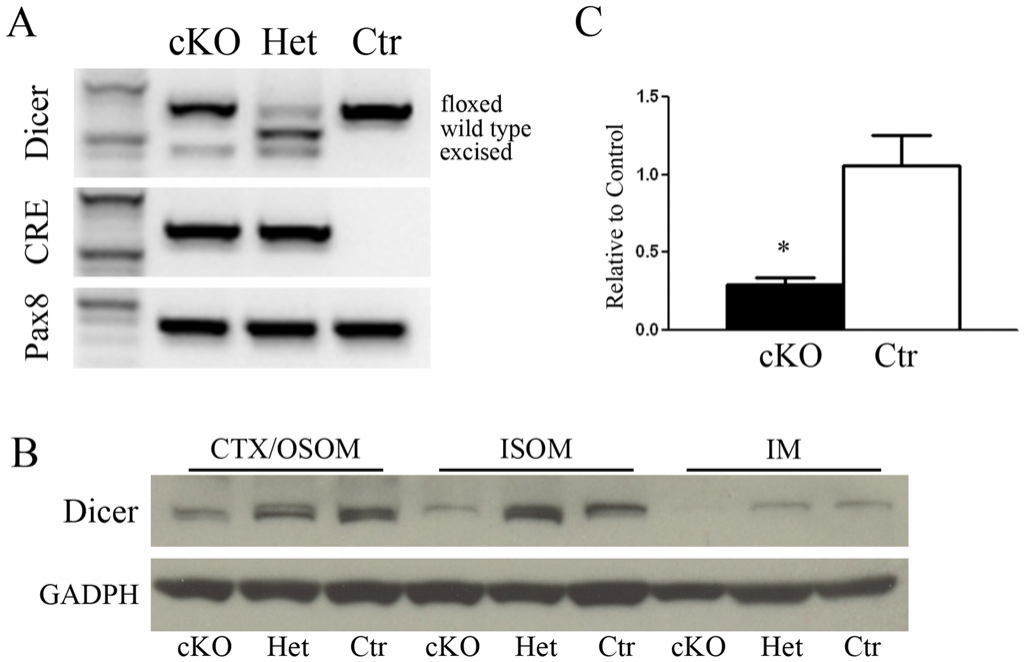
Dicer inactivation in mutant mice. Panel A shows the genotyping of Dicer, Cre and Pax8 genes in mice either homozygote (cKO) or heterozygote (Het) for Dicer and control mice (Ctr). PCR of renal cortical tissues at P50 shows the Dicer allele floxed, wild type and excised forms. Cre is detected only in homozygous and heterozygous cKO mice as expected, while Pax8 is present constitutively in all the groups. Panel B shows western blot analysis of renal zones Cortex/OSOM, ISOM and IM samples from 50 day old mice. A reduced abundance of Dicer in cKO mice can be observed in all the zones. Normalization was carried out with GAPDH. Panel C shows qPCR analysis of Dicer mRNA. A substantial reduction of Dicer can be seen in cKO mice compared to Ctr. Data are expressed as mean ± sem.

In order to study the overall function of the miRNA in the kidney, a conditional knockout (cKO) mouse model for Dicer in Pax8 expressing cells was generated (Dicer^Flox/Flox^;Pax8^Cre/+^). PCR of renal cortical tissues at P50 showed a different set of Dicer genes according to the expected cleavage from Cre-recombinase (Fig. 1A).

Residual Dicer protein expression in the renal zones has been evaluated by immunoblotting. In Dicer cKO (Dicer^Flox/Flox^;Pax8^Cre/+^) mice the expression of Dicer is lower compared to heterozygous (Dicer^Flox/+^;Pax8^Cre/+^) and Ctr mice (Dicer^Flox/Flox^;Pax8^+/+^) in CTX/OSOM, ISOM and IM (Fig. 1B). Dicer mRNA in Cre positive mouse is shown in Fig. 1C, confirming an overall reduction of the mRNA.

### Dicer cKO mice develop severe renal failure, progressively massive proteinuria and urinary concentrating defect

**Fig 2:**
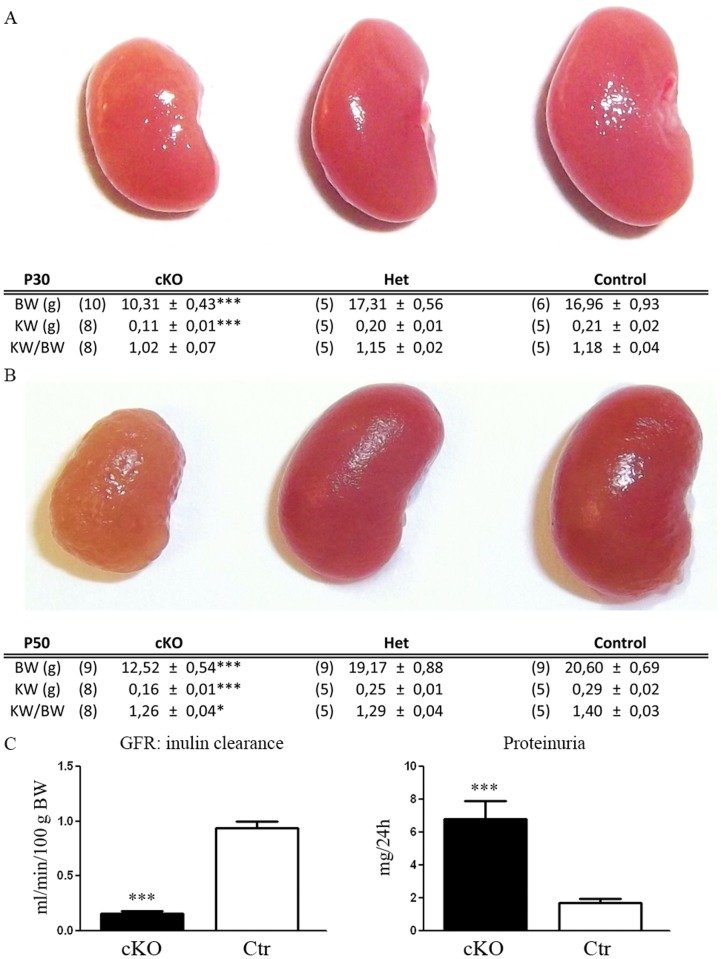
Dicer suppression induces alterations in renal morphology and function. Morphology of the kidneys in control (Ctr) and heterozygous (Het) or homozygous (cKO) Dicer cKO mice at P30 and P50 and their relative kidney (KW) and body (BW) weight. Panel A shows kidneys at P30 have a smaller size in cKO mice compared to the other group. This is correlated with low BW (similar KW/BW). In panel B, at P50, the kidneys of the Dicer cKO mice have a rough and pale structure compared to the other groups. Differences in KW/BW ratio in cKO mice suggest that the development of renal failure is coupled with morphological alteration. Heterozygous mice (Het) do not show any differences from Ctr mice. Panel C shows the glomerular filtration rate (GFR) measured by the clearance of inulin (left) and the urinary protein abundance in a 24h collection (right). At P50, cKO mice present a significantly altered GFR and proteinuria compared to Ctr. Data are expressed as mean ± sem; n power is expressed in brackets.

Dicer cKO mice do not show any macroscopic differences from control littermates at birth; however, they show failure to thrive and a higher mortality rate [16]. At P30, they present lower body weight (BW) and kidney weight (KW) (Fig. 2A) with respect to Ctr mice, but there were no differences in the relative ratio KW/BW, suggesting that the progression of KW is linked to BW. However, at P50, in addition to a lower KW, the ratio KW/BW is also significantly lower in Dicer cKO mice (Fig. 2B). At this stage, the kidneys of the cKO mice have a rough surface and a paler colour (Fig. 2B). These gross morphological alterations are associated with a lower GFR (ml/min/100g BW) as measured by Inulin clearance (cKO 0,15 ± 0,03, n:5 and CTR 0,94 ± 0,04 n:5; p<0,001) and a massive proteinuria (mg/24 h) (cKO 6,76 ± 0,87 n:6 and CTR: 1,17 ± 0,26 n:7; p = 0,0001) (Fig. 2C). Proteinuria mainly consists of albuminuria (S1 Fig.) and is already significantly higher in cKO mice at P30 (cKO 2,43 ± 0,37 n:8 and CTR 1,13 ± 0,17 n:4 p = 0,041).

**Fig 3:**
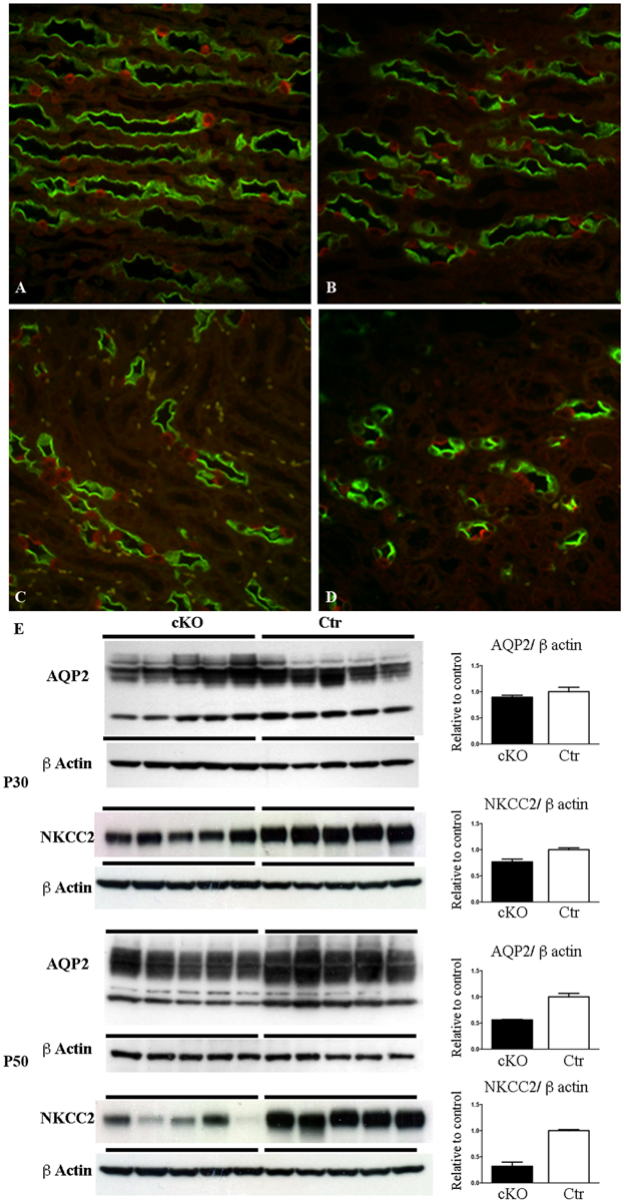
Dicer suppression is associated with downregulation of AQP2, NKCC2 and lower density of collecting ducts. Representative pictures of the inner stripe of the outer medulla of control (A, C) and Dicer cKO (B, D) mice double labeled with anti-AQP2 (green) and anti-H^+^ATPase (red) antibodies. At P50, Dicer cKO mice (D) showed no alteration in the distribution pattern of principal (AQP2 +) and intercalated cells (H^+^ATPase +), but a lower density of collecting ducts compared to the controls (C). (A-D) Magnification 40X. (E) Western blot analysis for AQP2 and NKCC2 expression at P30 (upper) and P50 (lower) of samples of IM and ISOM, respectively. AQP2 expression is significantly downregulated in Dicer cKO mice compared to Ctr at P50. NKCC2 expression is significantly downregulated in Dicer cKO mice compared to Ctr at P30 and P50. Data are expressed as mean ± sem; n power is 5 vs 5. *** is for p value < 0.001.

cKO mice progressively present an impairment of urinary concentrating ability. At P30 urinary osmolality is lower in the cKO mice compared with the control, with no changes in urinary output (Table 1) or in collecting duct (CD) distribution or variation of AQP2 abundance (Fig. 3). However, at P30, cKO mice presented a lower abundance of NKCC2 than Ctr mice in samples of inner stripe of outer medulla (ISOM). At P50 the urinary concentrating defect becomes evident (Table 1) and is associated with downregulation of AQP2 and NKCC2 protein expression (Fig. 3E). Double labeling for principal (AQP2+) and intercalated cells (H^+^-ATPase+) showed no alteration in the cellular distribution at both P30 and P50 (Fig. 3 A-D). However, at P50 the density of collecting ducts in Dicer cKO mice (Fig. 3D) is lower than Ctr (Fig. 3C), as investigated in the ISOM, the renal zones where the medullary rays converge. Morphological reduction in the numbers of CD and downregulation of AQP2 confirm impairment in urinary concentration ability. No hydronephrosis has been detected in Dicer cKO mice.

**Table 1:** 

		P30			
		cKO		Ctr0	
Urine Output	μl/min/gBW	(10)	44.57±7.67	(5)	49.07±6.98
Urine Osmolality	mOsm/Kg H2O	(5)	1169±160**	(4)	2228±221
Water Intake	ml/gBW	(10)	0.58±0.04**	(6)	0.42±0.01
Food Intake	g/gBW	(10)	0.25±0.02	(6)	0.27±0.02
		**P50**			
			**cKO**		**Ctr0**
Urine Output	μl/min/gBW	(7)	176.82±20.45***	(9)	52.33±8.65
Urine Osmolality	mOsm/Kg H2O	(5)	750±58.3***	(6)	2207±188
Water Intake	ml/gBW	(7)	0.67±0.04***	(9)	0.36±0.05
Food Intake	g/gBW	(7)	0.23±0.03	(9)	0.22±0.02

Data are expressed as mean ± sem; n is expressed in brackets.

** is for p <0.01;

*** is for <0.001

### Suppression of Dicer leads to a glomerulocystic phenotype

**Fig 4:**
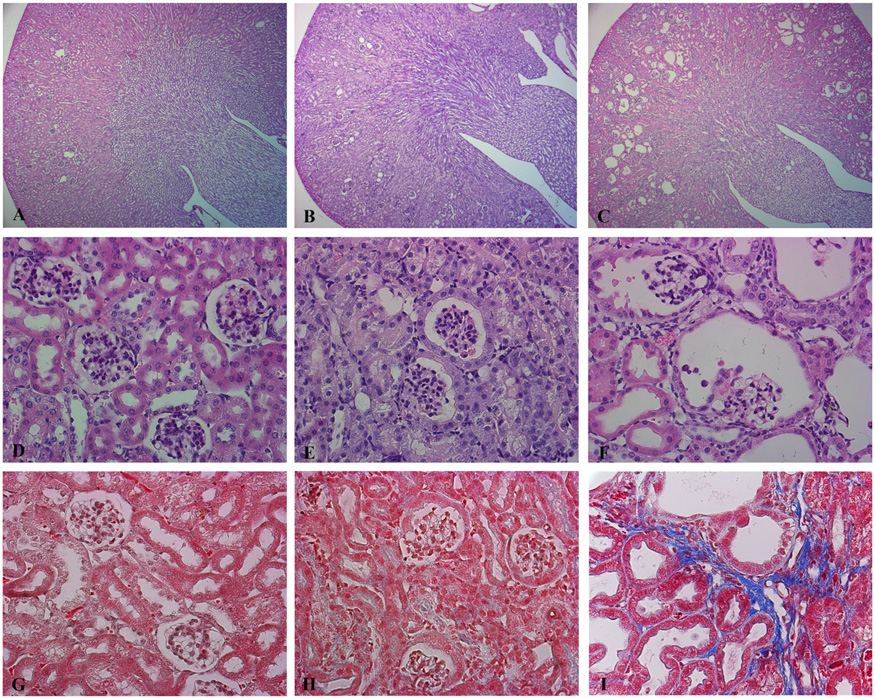
Suppression of Dicer leads to a glomerulocystic disease. Representative pictures of hematoxylin and eosin (A-F) and Masson’s Trichrome (G,I). Staining of Ctr (A, D, G), Dicer cKO kidneys at P30 (B, E, H) and Dicer cKO kidneys at P50 (C, F, I). Dicer induced cyst formation is limited to the cortex (C). Dicer cKO mice present a glomerulocystic phenotype and tubular dilatation at P50 (F). These alterations are not so prominent at P30, where only sites of dilatation in Bowman capsule can be sporadically seen (E). Interstitial fibrosis is evident in Dicer cKO mice at P50 (I). (A, B, C) Magnification 5X. (D- I) Magnification 40X.

**Fig 5:**
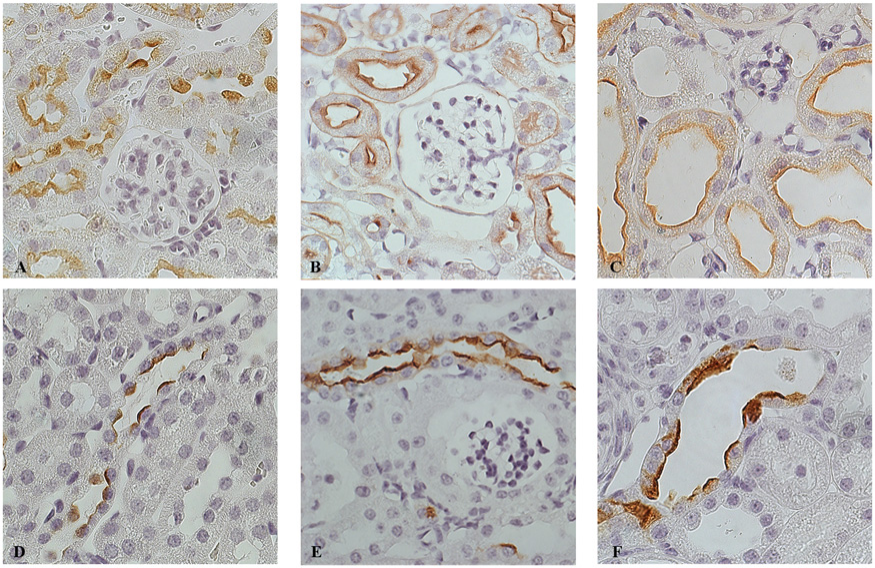
Tubular dilatation mainly involves the distal segments of the nephron. Representative pictures of renal cortex from Ctr mice (A, D) and Dicer cKO at P30 (B, E) and at P50 (C, F) mice stained with LAH (Lectin from Arachis Hypogaea) (A- C) and AQP2 (D- F). Dilated tubules in P50 Dicer cKO mice are positive for LAH (C) and AQP2 (F), reflecting their origin from distal convolute tubules, connecting and collecting ducts. (A-F) Magnification 63X.

At P50 Dicer cKO mice present cortical cysts (Fig. 4C). Cysts are absent in the outer and inner medulla. They mainly originate from the renal corpuscles of the cortex. Together with a dilatation of the parietal cells of the Bowman capsule, the glomeruli progressively shrink (Fig. 4F) in a pattern similar to glomerulocystic disease. The epithelium lining the cyst flattens and interstitial fibrosis develops (Fig. 4I). This severe morphological alteration correlates with the impairment of the glomerular filtration rate (GFR). These alterations are not so prominent at P30, where only sites of dilatation can be sporadically seen (Fig. 4E). Tubular dilatations are mainly cortical and they progressively develop at P50. The epithelium lining the dilated structures in P50 cKO mice stains with Lectin from Arachis Hypogaea (LAH, a marker of CD and distal tubules) and AQP2, a specific marker of CD (Fig. 5C,F), while no immunoreactivity for the sodium phosphate cotransporter, NaPi-2a (a specific marker of the proximal tubule) or Tamm-Horsfall Protein (THP: expressed exclusively in the thick ascending limb) can be detected in dilated tubules (S2 Fig.).

### High cellular turnover and the loss of the primary cilium are associated with the development of cysts in Dicer cKO mice

**Fig 6:**
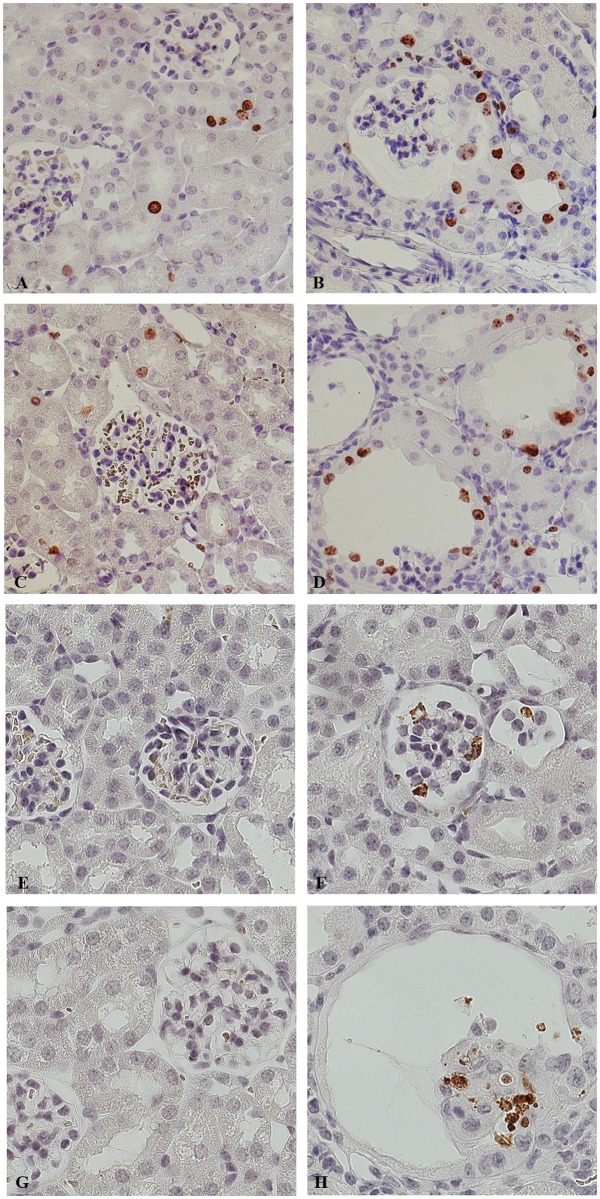
High cellular turnover precedes the development of cysts. Representative pictures of renal cortex from 30 (A, B, E, F) and 50 day old mice (C, D, G, H). Ctr (A, C, E, G) and Dicer cKO (B, D, F, H) renal sections were stained with anti-ki67 (marker of proliferation) (A-D) and anti-Cleaved Caspase 3 (marker of apoptosis) (E-H). Before developing the Dicer-induced glomerulocystic phenotype, the epithelium lining the dilating structure is already immunoreactive to the proliferation marker ki67 (B). Higher proliferation rate is evident also in dilated structure (D). Glomerular and tubular cells of Dicer cKO mice at both P30 and P50 (F, H) were positive for the apoptosis marker, Cleaved Caspase 3. (A-H) Magnification 40X.

Cellular proliferation rate has been investigated by immuno-staining with an anti-ki67 antibody. Cells labeled for anti-ki67 antibody are more widely detected in renal section of Dicer cKO mice than Ctr. The epithelium lining the border of dilating structures is frequently anti-ki67 immunoreactive. Therefore, the tubular epithelium and parietal cells of the renal corpuscles express ki67 protein in the phase of cyst growth, before flattening of the cystic epithelium. This is evident both at P30 (Fig. 6A-B) and P50 (Fig. 6C-D).

Apoptosis has been investigated by immuno-staining with an anti-Cleaved Caspase-3 antibody. In Dicer cKO mice, apoptotic cells are more frequent than in the Ctr group. Apoptosis is frequently seen in shedding cells in both the Bowman space and in the lumen of the tubules (Fig. 6E-F). This occurs more frequently at P50 (Fig. 6G-H). Taken together these data show a higher cellular turnover of the epithelium lining the cysts.

**Fig 7:**
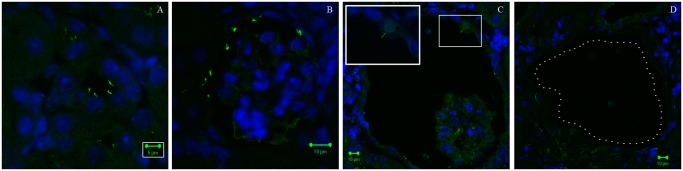
The development of the glomerulocystic phenotype is progressively associated with loss of primary cilium. Representative pictures of renal cortex from 50 days old Ctr (B) and Dicer cKO mice (A, C, D) stained for α-Acetylated Tubulin (green) and counterstained with Dapi (blue). Primary cilium is normally found in non-dilating tubules of Dicer cKO mice (A) and in the parietal cells of Ctr mice (B) at P50. However, presence of cilia is decreased progressively in dilating glomeruli (C) until disappearing in frank cysts (D). Bars = 5 μm (A); 10 μm (B-D).

The primary cilium seems to be not directly affected by the suppression of Dicer. At P30 primary cilia are normally present in both parietal and tubular epithelial cells (not shown), indicating no alterations at pre-cystic stages. In addition, at P50, Dicer cKO mice show the normal presence of the primary cilium in undilated tubules (Fig. 7A) to the same extent as the Ctr group (Fig. 7B). However, primary cilia are rare in the epithelium of dilating tubules and absent in enlarged cysts with flattened epithelium. The loss of primary cilia seems to be related to the cyst size, suggesting a progressive loss associated with their enlargement (Fig. 7C-D).

### Dysregulation of GSK3β/β-catenin, but not mTOR/S6, pathway is associated with the development of a glomerulocystic phenotype in Dicer cKO mice

Glomerulocystic morphological alterations are common in nephronophthisis and medullary cystic kidney disease. Several evidences show that these conditions are associated with dysregulation of the canonical and/or non-canonical Wnt-pathways.

**Fig 8:**
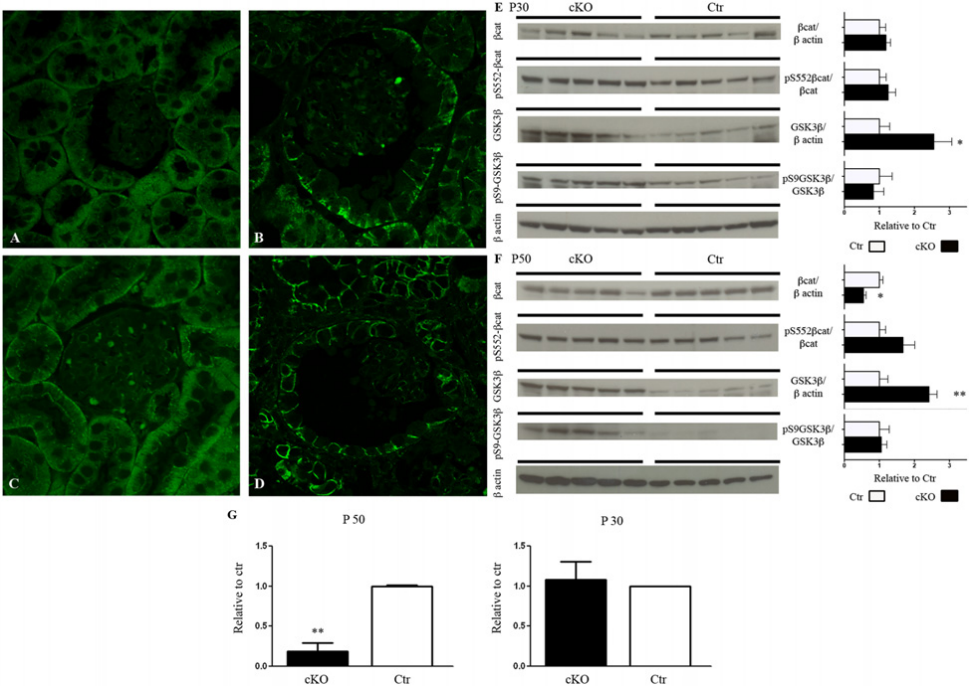
Alterations of GSK3β / β-catenin are associated with the development of the glomerulocystic phenotype. Representative pictures of renal cortex from 30 (A, B) and 50 days old (C, D) mice. Ctr (A, C) and Dicer cKO (B, D) sections were stained with anti β-catenin antibody. In Dicer cKO, β-catenin expression is limited to the basolateral domain in both tubules and parietal cells of the Bowman’s capsule, at both P30 and P50 (B-D). (A-D) Magnification 63X, zoom1.5. Panel E and F show immunoblotting of Cortex/OSOM samples of 30 (E) and 50 (F) days old mice. GSK3β expression is already upregulated at the pre-cystic stage (P30) in Dicer cKO mice (E) and this is persistent at P50 (F). Glomerular cyst formation is associated with loss of cytosolic β-catenin expression and general downregulation of cortical protein level (F). No significant changes were observed in the fraction of their main regulating phosphorylated forms. Panel G represent qPCR analysis of mRNA of β-catenin in 30 (right) and 50 days old mice (left). mRNA level of β-catenin is downregulated in Dicer-cKO mice at P50. Data are expressed as mean ± sem; n power is 5 vs 5. * is for p value < 0.05; ** is for p-value <0.01.

Here we show that, in Dicer cKO mice, the epithelium lining the cysts shows a lower expression of cytosolic but not membrane-associated β-catenin. This change occurs progressively, slight in dilating structures at P30 and more severe in frank cyst at P50 (Fig. 8B-D). Immunoblotting confirms a significant downregulation of β-catenin compared to control in P50 Dicer cKO mice (Fig. 8F), while at P30 no significant difference was detectable (Fig. 8E). Quantification of mRNA for β-catenin mirrors protein abundance (Fig. 8G). Change in the phosphorylation status of the β-catenin protein modulates enzyme activity; β-catenin phosphorylated at Ser552 (pS552 β-catenin) has been described as an active form [19]. Fractional expression of pS552 β-catenin over the total form is similar in cKO and Ctr mice at both P30 and P50 (Fig. 8E-F).

GSK3β regulates cytosolic abundance of β-catenin by promoting its proteasomal degradation, while GSK3β phosphorylation at Serin9 inhibits its activity [20]. Already after 30 days from birth, Dicer cKO mice show a significant upregulation of GSK3β, that persists at P50 (Fig. 8E-F). No significant variation in the pS9 GSK3β isoform over the total protein was detectable at time point. Thus, GSK3β upregulation precedes cytosolic β-catenin downregulation in Dicer cKO mice.

Since mTOR dysregulation is involved in several cystic disease models we further investigated mTOR expression in Dicer cKO mice. mTOR expression is downregulated in Dicer cKO mice at P30 when the structural abnormalities are not so prominent yet. No changes in pS2448 mTOR or its downstream regulator S6 and pS235–236 S6 are detectable. At P50, when the glomerulocystic phenotype is massive, no alteration in mTOR/S6 pathway can be seen (S3 Fig.).

## Discussion

Dicer is an important protein for miRNA maturation and function. miRNAs are involved in post-transcriptional regulation of several proteins, and their role is crucial for many organs. Deletion of Dicer is the most common approach to study the role of miRNAs in development and disease. However a total knock down approach is lethal for rodents [5] and so several cKO models have been created. In humans, constitutional haploinsufficiency for Dicer has been associated with a high frequency of PPB-FDS, and a renaming of PPB-FDS to Dicer syndrome has been proposed [11]. PPB-FDS patients or their family members carrying a truncating mutation often present renal nephroma and multinodular goiter. To investigate the underlying mechanisms involved in the renal phenotype of PPB-FDS we reproduced the two most frequent phenotypes of PPB-FDS by knocking down Dicer specifically in Pax8 expressing cells. The thyroid has been extensively investigated previously [16]; here we focus on the renal phenotype, showing that miRNAs dysregulation alters the GSK3β/β-catenin pathway and induces cystogenesis in Dicer cKO mice.

### Overall morphological and functional phenotype

Dicer cKO mice show a failure to thrive associated with a high mortality rate around 2 months of age. At P30, mice present a lower kidney weight (KW) compared with their littermates. This follows the overall reduction in body weight (BW). No major alterations in gross morphology are seen at this stage. The reduced KW is relative to BW (normal KW/BW ratio) resembling the effects of congenital hypothyroidism in humans [21]. Since at birth these mice have normal thyroid morphology and do not present evident differences from their littermates, lower growth rates could be ascribed, at least in part, to postnatal reduced thyroid hormone synthesis [16].

Investigation of 30 day old Dicer cKO mice showed tubular dilatation and rare cyst formation associated with an increased proteinuria. The changes in the morphological and functional structure of the kidneys become progressively more evident. A smaller size, rough and pale gross morphology is predominant at P50. At a microscopic level, cortical cysts and interstitial fibrosis reflect the functional finding of a decreased GFR. These features together with a large albuminuria suggest that renal failure is one of the main causes of death in Dicer cKO mice.

### Cyst development and pathogenesis

A progressive dilatation of the parietal cells lining the Bowman capsule and the collapse of the glomeruli reflect the pattern of glomerulocystic disease. Tubular dilatation involves mainly the distal segments of the nephron, including the collecting ducts, and is limited to the cortex.

The appearance of these morphological alterations is preceded by an increase in cellular turnover of their own epithelium, as seen in other models of cystogenesis [22]. In our model, we observed a progressive enlargement of the Bowman’s capsule associated with a loss of the primary cilia in large cysts. Since cilia are present normally in un-dilated, and in dilating, parietal cells in Dicer cKO mice, it is unlikely to be a direct effect of miRNA dysregulation. However, we cannot exclude an impairment of the function of the primary cilium that is secondary to miRNA dysregulation at the pre-cystic stage. This is in accordance with another ciliopathy, OFD-1 syndrome [23].

Cortical cysts arising from the parietal cells of Bowman’s capsule have been previously identified in Ksp/cre; Dicer^F/F^ mice [10]. Pax8 and Ksp are broadly expressed in the renal epithelium and mostly overlap in their expression domains [14]. Thus these cysts could be a direct consequence of the impairment of miRNA function in the parietal cells of Bowman’s capsule, distal nephron and collecting duct. The proximal tubule does not seem to contribute to cystogenesis in our model, as in others [10], even though we cannot exclude that Cre-Lox recombination in this segment is less efficient (both immuhistochemical and in-situ hybridization for Dicer was unsuccessful to rule out this possibility). The fact that some segments of the nephron are more susceptible to Dicer function seems to be endorsed by our study.

Patel V. *et al*. have shown that in Ksp/cre; Dicer^F/F^ mice the suppression of the miRNA200 family stimulates Pkd1 expression and consequently the development of cysts [10]. Here we show that dysregulation of the GSK3β/β-catenin, but not mTOR/S6 pathway is another crucial player for Dicer-dependent cyst development.

In Dicer cKO mice, β-catenin expression (both mRNA and protein level) is downregulated in the cortex of 50 days old mutant mice. β-catenin downregulation may be associated with the observed GSK3β upregulation and subsequent cyst development. This hypothesis is supported by a similar glomerulocystic pattern developed by β-catenin cKO mice in Pax8 expressing cells [24]. These mice, in addition to glomerular cysts, present with a urinary concentrating defect, suggesting a crucial role of β-catenin for the proper functioning of the collecting duct [24]. This is a further evidence of the crucial role of Wnt/β-catenin pathways in glomerulocystic diseases [25]. GSK3β upregulation could account also for mTOR downregulation in 30 days old mice, since its well known inhibitory effect on the expression of mTOR [26]. On this line, no variation of the mTOR downstream regulators S6 and pS6 could be detected in Dicer cKO mice.

Our data indicate that the link between miRNA dysregulation and β-catenin suppression may be mediated by the upregulation of GSK3β. GSK3β regulates soluble, cystosolic, β-catenin expression by promoting its proteasomal degradation, while phosphorylation in S9 of GSK3β mediates β-catenin stabilization [20].

Since GSK3β mRNA has multiple target sites for several members of the miRNA200 family (www.transgenescan.com) the suppression of miRNA200 family in Dicer cKO mice [10] could determine GSK3β cytosolic accumulation and therefore β-catenin inhibition. Upregulation of Pdk-1 is an additional mechanism promoting GSK3β upregulation. It has been shown in MDCK cells that the overexpression of Pdk-1 increases the activity of GSK3β, by reducing its S9-phosphorylation levels, and so modulating β-catenin expression [27]. Here we cannot see significant changes in p-S9GSK3β expression over total GSK3β, but we cannot exclude addition levels of interaction between GSK3β and Pdk-1.

### Development of urinary concentrating defect

Together with the development of renal cysts, Dicer cKO mice show a progressive urinary concentrating defect. A urinary concentrating defect is often associated with ciliopathy, such as in ADPKD patients [28].

Dicer cKO mice have a slight polydipsia and reduction in urinary osmolality associated with no morphological alteration of the renal medulla at P30, while an overt polydipsia, polyuria and lower urinary osmolality appear at P50 together with loss of CD and AQP2 downregulation. cKO mice present equal food intake compared with the control group at any studied stage (Tab-1), confirming that there is no influence of the osmolar intake on the urinary osmolality. At P30 cKO mice already present a lower abundance of the NKCC2 protein. Since NKCC2 is crucial for sodium and chloride reabsorption along the thick ascending limb of the Henle’s loop and so for the generation and the maintenance of the medullary interstitium to blood hyperosmolar gradient, this could suggest that a lower interstitial osmolar gradient precedes the development of the urinary concentration defect (P50).

The development of a urinary concentrating defect reveals a crucial role for Dicer in the CD, in line with a previous study where Dicer inactivation in HoxB7 expressing cells leads to congenital hydronephrosis and development of multiple cysts in the CD [8]. In this setting, GSK3β/β-catenin dysregulation could directly affect the epithelium of the CD. β-catenin is an early target of vasopressin response in principal cells [29]. Similar alterations in the β-catenin regulatory pathway have been associated with lithium-induced polyuria, an experimental model causing the development of renal cysts [30]. Finally, since in our cKO mice the concentrating ability is lost progressively and not acutely, we cannot exclude that it may be related, at least partly, to the development of renal failure.

## Conclusion

Here we showed that the integrity of the miRNAs system is essential to preserve the overall morphology and function of the renal corpuscles and collecting ducts in mouse kidneys. We showed that upregulation of total GSK3β occurs early in the Dicer cKO mice and could be responsible for β-catenin regulation and development of the glomerulocystic phenotype. This pathway appears crucial in the pathophysiology of renal cystic diseases and may be instrumental for our understanding of the mechanisms causing PPB-FDS (Dicer Syndrome).

## Supporting Information

S1 Fig Dicer-suppression induces albuminuria. Blue-Coomassie staining of SDS gel loaded with bovin serum albumin (BSA, positive control), urine samples from homozygous (cKO), heterozygous (Het), and control (Ctr) 50 day old mice. Protein bands are detected only in Dicer cKO mice and they are at the same molecular size of the BSA positive control.
(TIF)

S2 Fig Proximal tubules and Thick Ascending Limb are not affected by dilatation in Dicer cKO mice. Representative pictures of 50 day old control (A, C) and Dicer cKO (B, D) mice stained with anti-NaPi2a (green A, B) or anti Tamm-Horsfall protein (THP) (green C, D) and Dapi counterstain (blue). No tubular dilatation is seen in NaPi2a and THP positive tubules. (A-D) Magnification 63X.
(TIF)

S3 Fig Dicer dependent cystogenesis is not associated with mTOR/S6 activation. Immunoblotting of samples from renal cortex of P30 (panel A) and P50 (panel B) mice. The relative abundance of mTOR and its phosphorylated form S2448 together with S6 and its phophorylated form S235–236, is reported. Dicer cKO mice present lower abundance of mTOR at P30 compared with control mice. Data are expressed as mean ± sem; n power is 5 vs 5. * is for p value < 0.05.
(TIF)
