# Supplementary figures and images for: Selective Dicer Suppression in the Kidney Alters GSK3β/β-Catenin Pathways Promoting a Glomerulocystic Disease
Source: PLoS One. 2015 Mar 23;10(3):e0119142. doi: 10.1371/journal.pone.0119142 (PMC4370407; doi:10.1371/journal.pone.0119142)

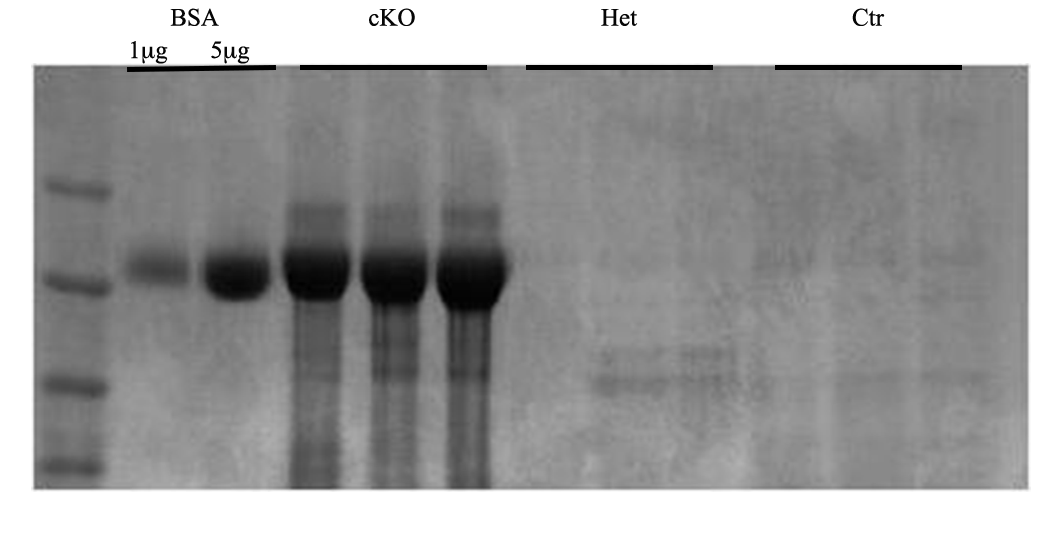

Supplement: S1 Fig — Blue-Coomassie staining of SDS gel loaded with bovin serum albumin (BSA, positive control), urine samples from homozygous (cKO), heterozygous (Het), and control (Ctr) 50 day old mice. Protein bands are detected only in Dicer cKO mice and they are at the same molecular size of the BSA positive control. (TIF) [file pone.0119142.s001.tif]

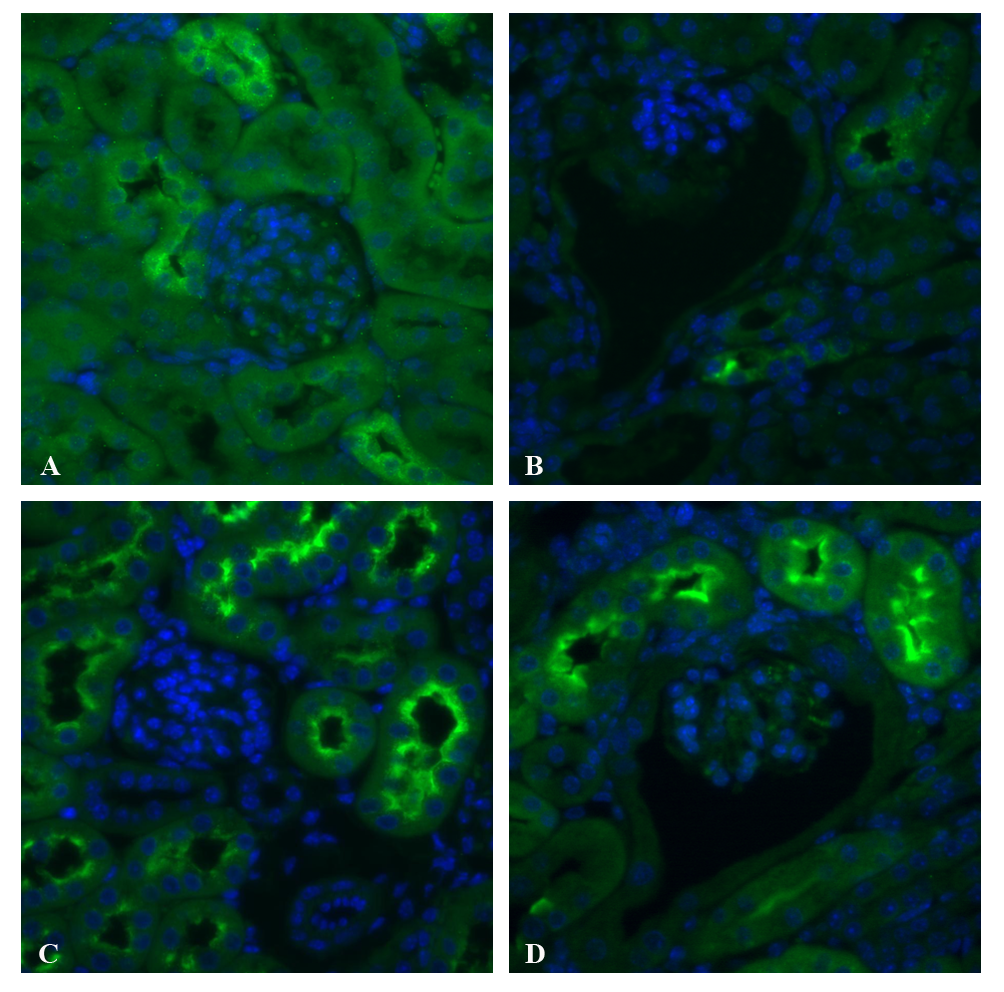

Supplement: S2 Fig — Representative pictures of 50 day old control (A, C) and Dicer cKO (B, D) mice stained with anti-NaPi2a (green A, B) or anti Tamm-Horsfall protein (THP) (green C, D) and Dapi counterstain (blue). No tubular dilatation is seen in NaPi2a and THP positive tubules. (A-D) Magnification 63X. (TIF) [file pone.0119142.s002.tif]

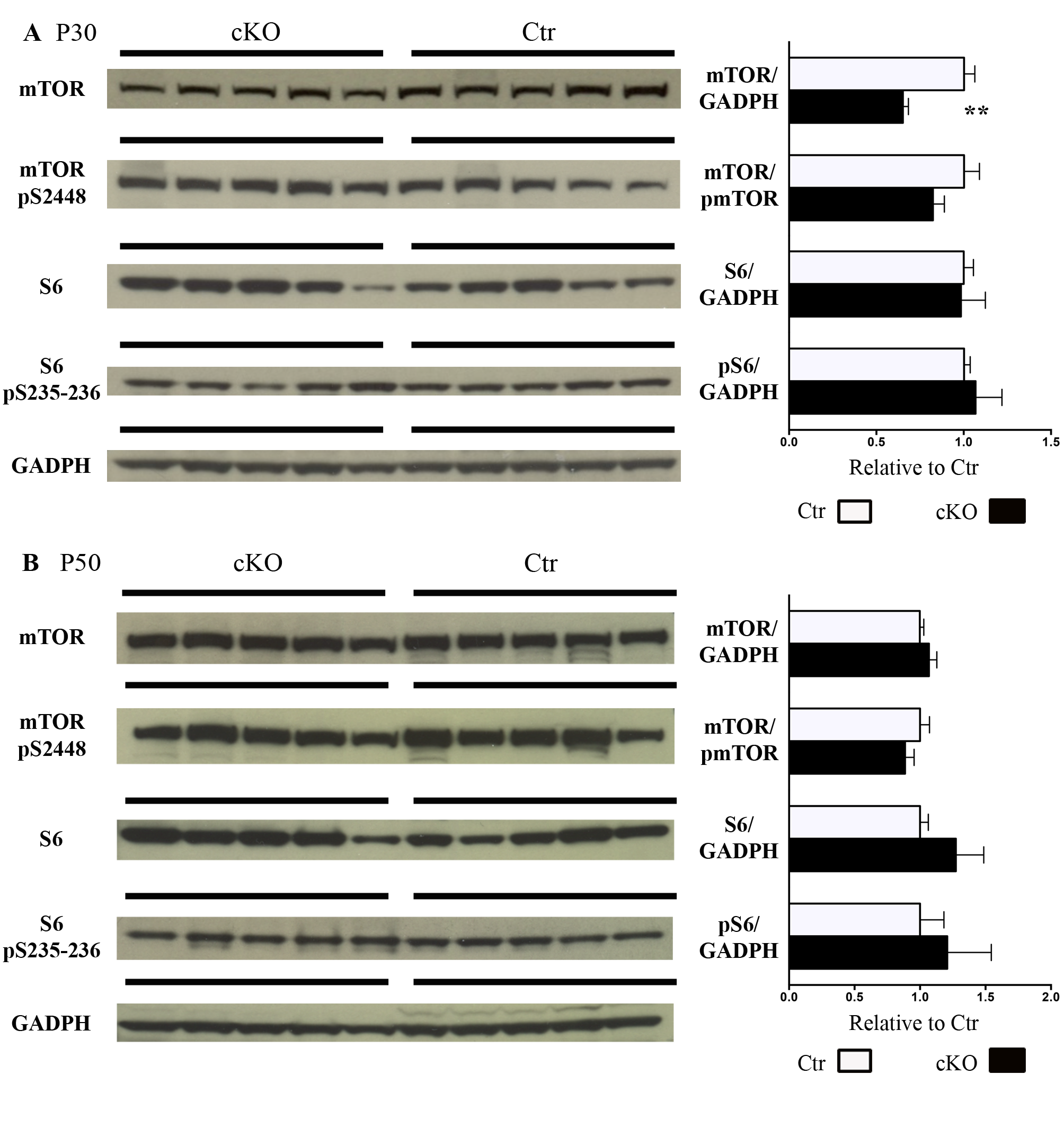

Supplement: S3 Fig — Immunoblotting of samples from renal cortex of P30 (panel A) and P50 (panel B) mice. The relative abundance of mTOR and its phosphorylated form S2448 together with S6 and its phophorylated form S235–236, is reported. Dicer cKO mice present lower abundance of mTOR at P30 compared with control mice. Data are expressed as mean ± sem; n power is 5 vs 5. * is for p value < 0.05. (TIF) [file pone.0119142.s003.tif]
